# The combination of initial markers to predict refractory *Mycoplasma pneumoniae* pneumonia in Chinese children: a case control study

**DOI:** 10.1186/s12931-020-01577-9

**Published:** 2021-03-22

**Authors:** Jun Wen, Yufei Su, Hongli Sun, Huiping Zhang, Hui Li

**Affiliations:** 1 Department of Neonatology, First Affiliated Hospital of Xi’an Jiaotong University, No. 227, Yanta West Road, Yanta district, Xi’an, Shaanxi 86-710061 People’s Republic of China; 2 Department of Emergency, Xi’an Children’s Hospital, Xi’an, Shaanxi 86-710061 People’s Republic of China; 3 Shaanxi Institute for Pediatric Diseases, Xi’an Children’s Hospital, Xi’an, Shaanxi 86-710003 People’s Republic of China; 4 Department of Neonatology, Xi’an Children’s Hospital, Xi’an, Shaanxi 86-710003 People’s Republic of China

## Abstract

**Objective:**

Thise study is aimed to identify the biomarkers for predicting refractory *Mycoplasma pneumoniae* pneumonia in Chinese children at the time of the hospital admission.

**Methods:**

The case control study retrospectively analyzed the clinical characteristics and laboratory results of Chinese pediatric patients presenting with common and refractory *Mycoplasma pneumoniae* pneumonia (CMPP and RMPP). Overall, there were 216 cases in the CMPP group and 88 cases in the RMPP group. Venous blood was collected, and serum ferritin (SF), lactate dehydrogenase (LDH), D-dimer, C-reactive protein (CRP), procalcitonin (PCT), neutrophil count/lymphocyte count (NLR), and other indexes were measured. A single factor analysis, an ROC curve analysis, and a logistic regression analysis were used to determine the independent risk factors of RMPP and find combination of initial markers for RMPP.

**Results:**

There were significant differences between the RMPP group and the CMPP group in mean SF (529.82 [357.86] vs. 147.22 [122.68] ng/mL), LDH (522.08 [389.08] vs. 286.85 [101.02] U/L), D-dimer (6.65 [5.66] vs. 1.46 [2.45] μg/mL), CRP (62.80 [52.15] vs. 19.03 [24.50] mg/L), PCT (0.80 [2.61] vs. 0.16 [0.44]) ng/mL, and NLR (4.14 [2.52] vs. 2.62 [1.55]), with *P* < 0.05 for each comparison. ROC cut-off values of the above indexes were 329.01 ng/mL, 375.50 U/L, 2.10 μg/mL, 43.08 mg/L, 0.08 ng/mL, and 2.96, respectively. The logistic regression analysis showed that SF, D-dimer, and CRP are independent risk factors to predict RMPP.

**Conclusion:**

SF, D-dimer, and CRP are statistically significant biomarkers to predict RMPP in Chinese children patients in the settings of pediatric emergency department.

## Introduction

*Mycoplasma pneumoniae* is one of the main pathogens of community-acquired pneumonia in children. *Mycoplasma pneumoniae* pneumonia (MPP) accounts for 10–40% of pneumonia in hospitalized children [1–3]. Refractory *Mycoplasma pneumoniae* pneumonia (RMPP) causes persistent fever and extra-pulmonary system involvement due to abnormal immune response, *Mycoplasma pneumoniae* resistance, and mixed infection (osteoarticular muscle and skin system, nervous system, mucosal system, cardiovascular system, blood system, urinary system, digestive system, etc.) [4–7]. There is no clear definition of RMPP. It is generally accepted that patients who have been treated with macrolide antibiotics for 7 or more days and still have same clinical signs/symptoms, persistent fever, and no changes in pulmonary imaging, may be considered as having RMPP [5, 8]. Severe cases can be complicated by pleural effusion, atelectasis, mediastinal air accumulation, pneumothorax, necrotizing pneumonia, and so on. Some children may develop respiratory distress and deteriorate rapidly requiring mechanical ventilation or extracorporeal membrane lung support [4]. Patients with MPP are often first seen in the emergency department, and early identification of RMPP remains challenging. Thus, it is necessary to identify specific and sensitive biochemical indicators for early diagnosis of RMPP.

Commonly used inflammatory indicators measured in routine blood tests are C-reactive protein (CRP) and procalcitonin (PCT). In some clinical studies, CRP has been shown to increase significantly in patients with RMPP, while other inflammatory indicators lack of sensitivity. Neutrophil/lymphocyte ratio (NLR)is a new indicator that has been used in more studies of inflammatory diseases in recent years, although its significance in RMPP is unknown. D-dimer can indicate whether the body is hypercoagulable, and serum ferritin (SF) can indicate whether macrophages are activated. Lactate dehydrogenase (LDH) is released during tissue damage and can thus indicate organ functional status.

The study aimed to identify the biomarkers for predicting refractory *Mycoplasma pneumoniae* pneumonia in Chinese children at hospital admission.

## Methodology

### Study population

From October 2019 to March 2020, 304 MPP patients were admitted to the emergency department of Xi'an Children's Hospital. Upon admission, all patients had signs and symptoms indicative of pneumonia, including fever, cough, abnormal lung auscultation, and a new infiltrate on chest radiograph [4, 5]. The diagnosis of an *M. pneumoniae* infection was based on positive serologic test results (*M. pneumoniae* anti-mp positive and antibody titer ≥ 1:160) and positive results for *M. pneumoniae* on polymerase chain reaction tests of nasopharyngeal secretions. Based on diagnostic criteria, which included aggravated clinical signs, persistent fever, and aggravated lung imaging despite treatment with macrolide antibiotics for 7 days, patients were divided into either the refractory *Mycoplasma pneumoniae* pneumonia (RMPP) group (88 patients) or the common *Mycoplasma pneumoniae* pneumonia (CMPP) group (216 patients) [4]. Patients with the following diseases were excluded in our study: congenital heart disease, chromosome disease, metabolic disease, immunodeficiency disease, blood tumor disease, bronchopulmonary dysplasia, nervous system dysplasia, and epilepsy, among others. In addition, patients were excluded if their medical history included neonatal respiratory distress syndrome, bacterial encephalitis, or other severe infectious diseases, such as severe acute respiratory syndrome coronavirus 2. Informed consents were obtained from all the guardians of the pediatric patients.

### Data collection

From October 2019 to March 2020, MPP patients were admitted to the emergency department of Xi'an Children's Hospital. Within 24 h after admission, patients in both the RMPP and the CMPP underwent tests for coagulation indicators, liver function, erythrocyte sedimentation rate (ESR), and CRP, PCT, myocardial enzymes, SF, and D-dimer. All data were analyzed retrospectively.

### Ethics

The study was approved by the ethics committee of the Xi’an Children’s Hospital, and patient data were analyzed anonymously. The authors asserted that all procedures contributing to this work comply with the ethical standards of the relevant national and institutional committees on human experimentation and with the Helsinki Declaration of 1975, as revised in 2008.

### Statistical analysis

The SPSS17.0 as used for data processing and statistical analysis. Mean ± SD ($\overline{x}$ ± s) representation was used for the measurement of normal distribution. A T test was used for inter-group testing. Categorical data were expressed as n (%), and a chi-square test was used for the comparison between groups. A single factor analysis was used to screen the indicators with significant differences. The characteristic ROC curve of positive index was drawn, the area under the curve (AUC) was calculated, and the predictive value of different biochemical indexes to RMPP was analyzed. An ROC curve analysis was used to determine the cut-off value for each index. A logistic regression analysis was used to determine the independent risk factors of RMPP and find the combination of initial markers for RMPP. The independent risk factors for RMPP were analyzed by multivariate logistic regression analysis and were considered statistically significant with *P* < 0.05. Using multivariate linear regression analysis to find the correlation between the independent risk factors.

## Results

### Comparison of baseline characteristics

There were no statistically significant differences between the RMPP group and CMPP group in demographics, including gender (*P* = 0.201) and age (*P* = 0.610), or in radiographic findings, including one lung pathology (*P* = 0.074), two lung pathology (*P* = 0.065), and lung consolidation (*P* = 0.937) (Table 1).

**Table 1:** Clinical characteristics of MPP and RMPP patients

Characteristic	CMPP (N = 216)	RMPP (N = 88)	t/Χ^2^	*P*
Mean age in years (SE)	6.07 (2.43)	6.47 (2.47)	− 1.28	0.201
Male, n (%)	106 (49.07)	46 (52.27)	0.26	0.610
Extra-pulmonary complication, n (%)	18 (8.33)	25 (28.40)	25.81	0.000
Pleural effusion				
One-sided, n (%)	41 (18.98)	45 (51.14)	31.87	0.000
Two-sided, n (%)	5 (2.31)	8 (9.09)	5.45	0.020
Radiological imaging				
One lung pathology, n (%)	164 (75.93)	58 (65.90)	3.18	0.074
Two lung pathology, n (%)	47 (21.75)	28 (31.82)	3.40	0.065
Lung consolidation, n (%)	5 (2.31)	2 (2.17)	0.006	0.937
Hospital stay (days)	7.77 ± 2.32	12.83 ± 5.22	− 11.69	0.000

*MPP* *Mycoplasma pneumoniae* pneumonia, *CMPP* Common *Mycoplasma pneumoniae* pneumonia, *RMPP* refractory *Mycoplasma pneumoniae* pneumonia, *SE* standard error

There were statistically significant differences (*P* < 0.05) between the RMPP group and the CMPP group in other disease characteristics. In the RMPP group 28.40% of patients experienced an extra-pulmonary complication, compared to 8.33% of patients in the CMPP group. One-sided pleural effusion was observed in 51.14% of the RMPP group and 18.98% of patients in the CMPP group, and two-sided pleural effusion was found in 9.09% of the RMPP group and 2.31% of the CMPP group.

### Comparison of clinical characteristics and laboratory results

The RMPP and control groups were admitted for 24 h to compare clinical symptoms, biochemistry levels, and routine blood markers. The duration of fever, the duration of cough, and the levels of fibrinogen, PCT, ESR, SF, LDH, CK-MB, cholesterol (CHO), aspartate aminotransferase (AST), alanine aminotransferase (ALT), neutrophil granulocyte count (NEP), NLR, and CRP were significantly higher in the RMPP group compared to the CMPP group (*P* < 0.05). Albumin was not significantly different between these two groups (*P* = 0.98) (Table 2).

**Table 2:** Clinical symptoms and laboratory characteristic of MPP and RMPP patients

Index	CMPP group	RMPP group	t/Χ^2^	*P*
Fever (days)	8.05 ± 3.99	9.03 ± 4.17	− 2.43	0.016
Cough (days)	8.26 ± 4.77	10.02 ± 5.28	− 2.82	< 0.01
PT (s)	14.14 ± 0.96	14.22 ± 10.08	− 0.46	0.65
APTT (s)	39.15 ± 2.65	37.24 ± 2.08	0.81	0.42
FIB (g/L)	3.87 ± 0.89	4.44 ± 1.09	− 4.71	< 0.01
TT (seconds)	17.10 ± 1.27	16.04 ± 1.36	0.76	0.45
PCT (ng/mL)	0.16 ± 0.44	0.80 ± 2.61	− 3.05	< 0.01
ESR (mm/h)	44.95 ± 24.22	58.03 ± 30.13	− 3.93	< 0.01
SF (ng/mL)	147.22 ± 122.68	529.82 ± 357.86	− 13.54	< 0.01
D-dimer (μg/mL)	1.46 ± 2.45	6.65 ± 5.66	− 4.35	< 0.01
LDH (U/L)	286.85 ± 101.02	522.08 ± 389.08	− 8.24	< 0.01
LDH-1 (U/L)	49.14 ± 29.61	55.15 ± 14.54	− 1.80	0.07
CK (U/L)	83.46 ± 81.40	209.85 ± 597.36	− 3.05	< 0.01
CK-MB (U/L)	19.71 ± 12.62	48.23 ± 226.65	− 1.83	0.07
TP (g/L)	42.12 ± 38.52	31.91 ± 4.25	2.46	0.01
ALB (g/L)	63.79 ± 4.82	63.71 ± 41.22	0.03	0.98
GLB (g/L)	27.59 ± 14.78	29.74 ± 23.39	− 0.95	0.34
CHO (mmol/L)	3.50 ± 0.70	3.06 ± 0.76	4.76	< 0.01
ALT (U/L)	22.09 ± 47.09	43.14 ± 49.45	− 3.43	< 0.01
AST (U/L)	30.69 ± 21.64	53.86 ± 44.73	− 6.00	< 0.01
WBC (10^9^/L)	10.27 ± 37.17	9.97 ± 4.60	0.07	0.94
LYM (10^9^/L)	2.83 ± 10.25	2.18 ± 1.56	0.58	0.56
NEP (10^9^/L)	5.07 ± 4.80	6.88 ± 3.91	− 3.14	< 0.01
NLR	2.62 ± 1.55	4.14 ± 2.52	− 6.40	< 0.01
HGB (g/L)	125.22 ± 76.04	115.93 ± 12.21	1.14	0.26
PLT (10^9^/L)	325.48 ± 106.48	327.53 ± 151.77	− 0.134	0.89
PM	32.91 ± 13.25	33.09 ± 19.19	− 0.09	0.93
CRP (mg/L)	19.03 ± 24.50	62.80 ± 52.15	− 7.54	< 0.01

*RMPP* refractory *Mycoplasma pneumoniae* pneumonia, *CMMP* common *Mycoplasma pneumoniae* pneumonia, *PT* prothrombin time, *APTT* activated partial prothrombin time, *TT* clotting time, *ESR* erythrocyte sedimentation rate, *PCT* procalcitonin, *SF* serum ferritin, *LDH* lactate dehydrogenase, *LDH1* lactate dehydrogenase isoenzymes, *CK* creatine kinase, *CK-MB* creatine kinase isoenzymes, *TP* total protein, *ALB* albumin, *GLB* globulin, *CHO* cholesterol, *ALT* alanine aminotransferase, *AST* aspartate aminotransferase, *WBC* leucocyte count, *LYM* leukomonocyte count, *NEP* neutrophil granulocyte count, *NLR* neutrophil count/lymphocyte count ratio, *HGB* hemoglobin concentration, *PLT* platelet count, *PM* platelet count/mean platelet volume, *CRP* C-reactive protein

### Predictive values of the independent correlation factors for patients with RMPP

**Fig. 1:**
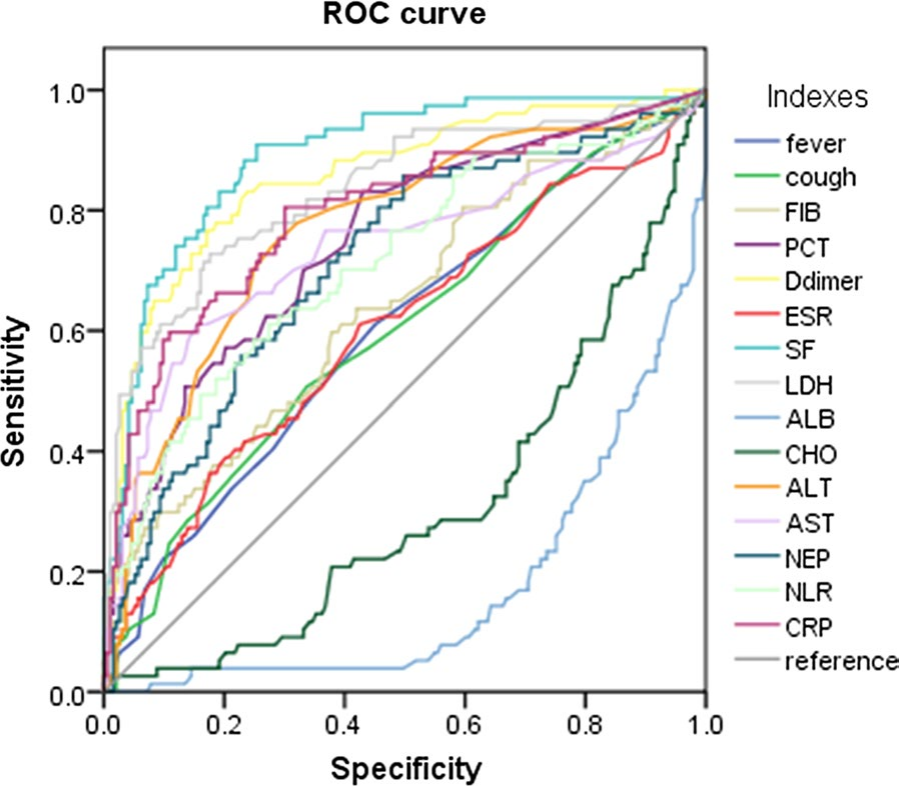
ROC curve. SF, D-dimer, LDH, and CRP had the highest AUC values (> 0.80), although ALT, PCT, AST, NLR, NEP, FIB, CK-MB, duration of fever, and duration of cough also achieved predictive significance. The AUCs of albumin and cholesterol, however, were < 0.50, which lacks predictive significance. *FIB* fibrinogen, *PCT* procalcitonin, *ESR* erythrocyte sedimentation rate, *LDH* lactate dehydrogenase, *ALB* albumin, *CHO* cholesterol, *ALT* alanine aminotransferase, *AST* aspartate aminotransferase, *NEP* neutrophil granulocyte count, *NLR* neutrophil count/lymphocyte count, *CRP* C-reactive protein, *D dimer* D-dimer, *SF* serum ferritin

ROC curve indicators with AUC > 0.50 suggests good prediction. SF, D-dimer, LDH, and CRP had the highest AUC values (> 0.80), although ALT, PCT, AST, NLR, NEP, FIB, CK-MB, duration of fever, and duration of cough also achieved predictive significance. The AUCs of albumin and cholesterol, however, were < 0.50, which lacked predictive significance The following cut-off values were used to predict RMPP: duration of fever > 7.5 days, duration of cough > 8.5 days, FIB > 2.10 g/L, PCT > 0.08 ng/mL, ESR > 69 mm/h, D-dimer > 2.1 μg/mL, SF > 329 ng/mL, LDH > 375 U/L, CK-MB > 52 U/L, ALT > 16.5 U/L, AST > 36.5 U/L, NEP > 4.5 × 10^9^/L, NLR > 2.96, and CRP > 43 mg/L (Table 3 and Fig. 1).

**Table 3:** Predictive values of the independent correlation factors for patients with RMPP

Index	AUC	95% CI	*P*	Cut-off value	Sensitivity %	Specificity%
Duration of fever	0.60 ± 0.04	0.53, 0.68	< 0.05	7.50	61.45	50.95
Duration of cough	0.60 ± 0.04	0.53, 0.69	< 0.05	8.50	53.01	66.83
FIB	0.65 ± 0.04	0.58, 0.73	< 0.01	2.10	79.75	81.86
PCT	0.75 ± 0.03	0.69, 0.82	< 0.01	0.08	80.72	57.42
ESR	0.61 ± 0.04	0.53, 0.68	< 0.01	68.50	39.76	83.17
D-dimer	0.87 ± 0.02	0.82, 0.92	< 0.01	2.10	79.01	81.86
SF	0.90 ± 0.02	0.86, 0.94	< 0.01	329.01	67.09	93.13
LDH	0.84 ± 0.03	0.79, 0.90	< 0.01	375.50	74.68	82.84
CK-MB	0.64 ± 0.04	0.56, 0.71	< 0.01	21.50	52.71	73.76
ALB	0.17 ± 0.03	0.11, 0.22	< 0.01	28.70	77.10	2.98
CHO	0.32 ± 0.04	0.25, 0.38	< 0.01	5.05	2.50	99.02
ALT	0.77 ± 0.03	0.71, 0.83	< 0.01	16.50	78.48	67.15
AST	0.73 ± 0.04	0.66, 0.80	< 0.01	36.50	60.24	81.26
NEP	0.69 ± 0.04	0.62, 0.76	< 0.01	4.50	72.94	58.69
NLR	0.72 ± 0.04	0.65, 0.79	< 0.01	2.96	63.86	73.27
CRP	0.81 ± 0.03	0.74, 0.87	< 0.01	43.08	62.03	82.73

*RMPP* refractory *Mycoplasma pneumoniae* pneumonia, *AUC* area under the curve, *CI* confidence interval, *FIB* fibrinogen, *PCT* procalcitonin, *ESR* erythrocyte sedimentation rate, *SF* serum ferritin, *LDH* lactate dehydrogenase, *CK-MB* creatine kinase isoenzymes, *ALB* albumin, *CHO* cholesterol, *ALT* alanine aminotransferase, *AST* aspartate aminotransferase, *NEP* neutrophil granulocyte count, *NLR* neutrophil count/lymphocyte count, *CRP* C-reactive protein

### Logistic regression analysis

A logistic regression analysis was performed on the indexes with predictive significance based on ROC curve analysis. The results showed that D-dimer, SF, and CRP were independent risk factors for RMPP (*P* < 0.05). The odds ratio values were 1.16, 1.00, and 1.02, respectively, as shown in Table 4.

**Table 4:** Stepwise logistic regression analysis for the related factors predicting the RMPP

Index	β	SE (β)	Wald Χ^2^	*P*	OR	95% CI
D-dimer	0.15	0.07	5.46	0.02	1.16	1.03, 1.32
SF	0.01	0.001	8.03	0.01	1.00	1.00, 1.01
CRP	0.02	0.01	6.66	0.01	1.02	1.00, 1.03

*RMPP* refractory *Mycoplasma pneumoniae* pneumonia, *SE* standard error, *OR* odds ratio, *CI* confidence interval, *SF* serum ferritin, *CRP* C-reactive protein

### Multivariate linear regression analysis

**Fig. 2:**
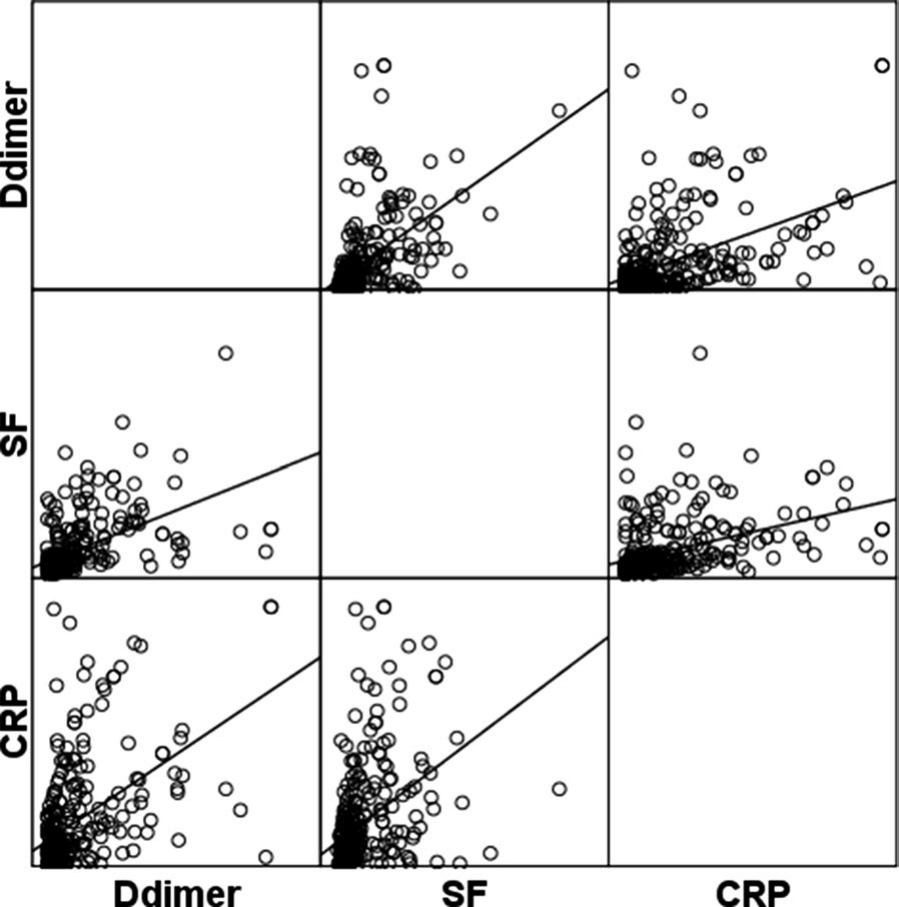
Scatter diagram. There was a positive linear correlation between SF, D-dimer and CRP. When one of them increased, so did any of the other two. *CRP* C-reactive protein, *D dimer* D-dimer, *SF* serum ferritin

A multivariate linear regression analysis was carried out for the independent risk factors obtained by logistic regression analysis. D-dimer was selected as the dependent variable, SF and CRP as the independent variables. Multivariate linear regression model analysis showed that the R was 0.37, indicated that the inclusion of positive indicators could explain 37% of the D-dimer variability, while the Dubin–Watson index was 1.63, and indicated slight non-independence, but had little effect on the accuracy of the regression results, as shown in Table 5. The results of multiple linear regression analysis showed that there was a positive correlation linear relationship between SF, CRP, and D-dimer, as shown in Tables  5, 6 and Fig. 2.

**Table 5:** Linear regression model for D-dimer

R	R party	Adjusted R party	Errors in standard estimates	Sabine Watson
0.61	0.37	0.37	3.50	1.63

**Table 6:** Results of multiple factor linear regression analysis

Index	β	SE (β)	t	*P*	Capacity	VIF
	0.204	0.297	0.69	0.492		
SF	0.036	0.006	6.35	< 0.01	0.83	1.21
CRP	0.006	0.001	7.68	< 0.01	0.83	1.21

## Discussion

*M. pneumoniae* is one of the main pathogens of community-acquired pneumonia in children. *M. pneumoniae* infection has been previously considered as semi-automatic (what does it mean). In recent years, more studies show that *M. pneumoniae* can led to death [5, 9, 10], and reports that the incidence of RMPP are increasing [5, 11–13]. It is still challenging to diagnose RMPP. The pathogenic mechanism of RMPP is very complex, which mainly includes both the direct lung cell injury and the immune response injury. Its pathological changes are interstitial pneumonia and, occasionally, bronchopneumonia, which is a primary atypical pneumonia that is spread through droplets. Recently, complications associated with RMPP have included necrotizing pneumonia, Stevens–Johnson syndrome, myocardial damage, endocarditis, encephalitis, meningitis, organ and peripheral artery embolism, liver dysfunction, and autoimmune hemolysis [4, 14, 15]. Long-term complications of RMPP include bronchiectasis, occlusive bronchitis, and unilateral transparent lung [14]. Thus, early identification of RMPP is particularly important for the long-term health of patients.

Beginning in the fall of 2019, the visits of MPP patients in our emergency department gradually increased, peaking between 2019 and January 2020. Most of the patients were diagnosed in the outside hospital, but transferred to our hospital due to serious complications. We retrospectively applied RMPP diagnostic criteria to 304 cases of MPP [4]. After considering other inclusion and exclusion criteria, we identified 88 cases of RMPP, the rest cases served as the CMPP group. There were no significant differences in age and sex between the two groups (P > 0.05).

RMPP early symptoms are mainly persistent high fever and cough. However, lung auscultation is often normal in this group. Therefore, RMPP is easily misdiagnosed as common cold or influenza. MPP should be considered if children around the age of five suffer from persistent high fever and severe coughing. Some studies have showed that high fever is an independent risk factor for RMPP [16, 17]. A single-factor analysis of the duration of fever and cough, respectively, upon admission was also included in this study, and significant differences were found (*P* < 0.05). While an ROC curve analysis revealed that a diagnosis of RMPP was more likely when the duration of fever and cough was greater than 7.5 days and 8.5 days, respectively [16], logistic regression analysis showed that these factors were not independent risk factors for RMPP.

Due to the risk of myocardial damage and liver damage associated with RMPP, a univariate analysis of the myocardial enzyme spectrum and liver function index was performed. We found that the serum levels of albumin and cholesterol were not significantly different between the RMPP and the CMPP groups, but levels of LDH, CK-MB, AST, and ALT were significantly different. The ROC curve analysis showed that LDH, CK-MB, AST, and ALT can be predictive of RMPP, but these indicators were not considered independent risk factors by regression analysis. Some studies have shown that LDH is an independent risk factor for RMPP, with a cut-off value 417 U/L considered predictive [17–21]. Our study found that an LDH cut-off value of 375 U/L LDH was predictive, with an AUC of 0.84 and specificity and sensitivity of 74.68% and 82.84%, respectively, but was not an independent risk factor for RMPP, so we think LDH can’t be a combined biomarker for predicting RMPP.

Blood cell analysis is often the first test in MPP diagnosis. However, it has not been shown to assist in RMPP early diagnosis. Cheng et al. showed that neutrophil ratio can be a significant predictor of RMPP, with a cut-off value of 68.6% [22]. In this study, single factor analysis of routine blood indexes showed that the values of NEP and NLR in the RMPP group were significantly higher than those of the MPP group. The AUCs were 0.70 and 0.72, and the cut-off values were 4.5 × 10^9^/L and 2.96, respectively. Logistic regression analysis of these two variables determined that they are not independent risk factors for RMPP. So, the combination of NEP and NLR may not be a good indicator to predict RMPP.

Among infectious diseases, PCT, CRP, ESR, SF is used as an inflammatory indicator to monitor the severity of infection, and we used these biomarkers to monitor MPP severity. Single factor analysis showed that the levels of PCT, ESR, SF, and CRP of the RMPP group were significantly higher than those of the CMPP group. The AUCs were 0.75, 0.61, 0.90, 0.81, respectively. As a result, we determined that the higher of SF, CRP, and PCT, the greater possibility of RMPP, particularly when their values are greater than 329 ng/mL, 43 mg/L, and 0.08 ng/mL, respectively. However, logistic regression analysis showed that only SF and CRP were independent risk factors for RMPP, with values of 1.00 and 1.02, respectively. Other studies have found similar results [14, 16, 18–21, 23–25]. We concluded that CRP and SF can be combined biomarkers to diagnoses RMPP.

D-dimer, a commonly used indicator of secondary hyperactive response to fibrinolysis, has been found increased significantly after MP infection [4, 26], and is more pronounced in RMPP [27]. Univariate analysis showed that D-dimer was significantly higher in the RMPP group than in the CMPP group in our study, and the AUC, truncation value, sensitivity, and specificity in the ROC were 0.87, 2.1 μg/mL, 79.1%, and 81.86%, respectively. Logistic regression analysis also showed that D-dimer is an independent risk factor of RMPP, with an OR value of 1.16. Therefore, D-dimer may be another biomarkers to diagnoses RMPP, in addition to SF and CRP.

## Conclusion

In summary, the combination of SF, CRP, and D-dimer, as the initial markers, can be used to predict RMPP. The higher their detected values, the more risk of having RMPP. This study has some limitations: the small sample size and a lack of external control. Further study will require a larger multi-institutional sample.

## Data Availability

The used raw data during this study are available from the corresponding author on reasonable request.

## Abbreviations

ALB: Albumin
ALT: Alanine aminotransferase
APTT: Activated partial prothrombin time
AST: Aspartate aminotransferase
CHO: Cholesterol
CK: Creatine kinase
CK-MB: Creatine kinase isoenzymes
GLB: Globulin
HGB: Hemoglobin concentration
LDH: Lactate dehydrogenase
LDH1: Lactate dehydrogenase isoenzymes
LYM: Leukomonocyte count
MPP: *Mycoplasma pneumoniae* Pneumonia
NEP: Neutrophil granulocyte count
NLR: Neutrophil count/lymphocyte count
PCT: Procalcitonin
PLT: Platelet count
PM: Platelet count/mean platelet volume
PT: Prothrombin time
RMPP: Refractory *Mycoplasma pneumoniae* pneumonia
SF: Serum ferritin
TT: Clotting time
WBC: Leucocyte count
